# A research framework for projecting ecosystem change in highly diverse tropical mountain ecosystems

**DOI:** 10.1007/s00442-021-04852-8

**Published:** 2021-01-30

**Authors:** Jörg Bendix, Nicolay Aguire, Erwin Beck, Achim Bräuning, Roland Brandl, Lutz Breuer, Katrin Böhning-Gaese, Mateus Dantas de Paula, Thomas Hickler, Jürgen Homeier, Diego Inclan, Christoph Leuschner, Eike L. Neuschulz, Matthias Schleuning, Juan P. Suarez, Katja Trachte, Wolfgang Wilcke, David Windhorst, Nina Farwig

**Affiliations:** 1 LCRS, Department of Geography, University of Marburg, Marburg, Germany; 2 Biodiversidad, Bosques y Servicios Ecosistemicos, Universidad Nacional de Loja, Loja, Ecuador; 3 Department of Plant Physiology and Bayreuth Centre of Ecology and Environmental Research, University of Bayreuth, Bayreuth, Germany; 4 Institute of Geography, Friedrich-Alexander-University (FAU) Erlangen-Nuremberg, Erlangen, Germany; 5 Animal Ecology, Department of Biology, University of Marburg, Marburg, Germany; 6 Institute for Landscape Ecology and Resources Management (ILR), Research Centre for BioSystems, Land Use and Nutrition (IFZ), Justus Liebig University, Giessen, Germany; 7 Senckenberg Biodiversity Climate Research Center (SBiK-F), Frankfurt am Main, Germany; 8 Institute for Ecology, Evolution and Diversity, Goethe University Frankfurt, Frankfurt am Main, Germany; 9 Department of Physical Geography, Goethe University Frankfurt, Frankfurt am Main, Germany; 10 Plant Ecology and Ecosystems Research, Albrecht Von Haller Institute for Plant Sciences, University of Göttingen, Göttingen, Germany; 11 Centre of Biodiversity and Sustainable Land Use (CBL), University of Göttingen, Göttingen, Germany; 12 Instituto Nacional de Biodiversidad, Sección Invertebrados, Quito, Ecuador; 13 Facultad de Ciencias Agrícolas, Universidad Central del Ecuador, Quito, Ecuador; 14 Departamento de Ciencias Biológicas, Universidad Técnica Particular de Loja, UTPL, Loja, Ecuador; 15 Institute for Environmental Sciences, Brandenburg University of Technology (BTU) Cottbus-Senftenberg, Cottbus, Germany; 16 Institute of Geography and Geoecology, Karlsruhe Institute of Technology (KIT), Karlsruhe, Germany; 17 Conservation Ecology, Department of Biology, University of Marburg, Marburg, Germany

**Keywords:** Biodiversity-land surface model, Functional traits, High mountains, Research framework, Response-effect-framework

## Abstract

Tropical mountain ecosystems are threatened by climate and land-use changes. Their diversity and complexity make projections how they respond to environmental changes challenging. A suitable way are trait-based approaches, by distinguishing between response traits that determine the resistance of species to environmental changes and effect traits that are relevant for species' interactions, biotic processes, and ecosystem functions. The combination of those approaches with land surface models (LSM) linking the functional community composition to ecosystem functions provides new ways to project the response of ecosystems to environmental changes. With the interdisciplinary project RESPECT, we propose a research framework that uses a trait-based response-effect-framework (REF) to quantify relationships between abiotic conditions, the diversity of functional traits in communities, and associated biotic processes, informing a biodiversity-LSM. We apply the framework to a megadiverse tropical mountain forest. We use a plot design along an elevation and a land-use gradient to collect data on abiotic drivers, functional traits, and biotic processes. We integrate these data to build the biodiversity-LSM and illustrate how to test the model. REF results show that aboveground biomass production is not directly related to changing climatic conditions, but indirectly through associated changes in functional traits. Herbivory is directly related to changing abiotic conditions. The biodiversity-LSM informed by local functional trait and soil data improved the simulation of biomass production substantially. We conclude that local data, also derived from previous projects (platform Ecuador), are key elements of the research framework. We specify essential datasets to apply this framework to other mountain ecosystems.

**Supplementary Information:**

The online version contains supplementary material available at 10.1007/s00442-021-04852-8.

## Introduction

Tropical mountains are biodiversity hotspots (Myers et al. 2000). At the same time, mountain ecosystems are vulnerable to environmental changes (Elsen and Tingley 2015). Losses of natural habitats lead to a rapid loss of species with their adaptations at low elevations and mountain-top extinctions that result from increasing temperatures (Steinbauer et al. 2018; Knoke et al. 2020). While changes in mountain biodiversity are increasingly documented (Peters et al. 2019), projecting the consequences for ecosystem functioning remains challenging, because of the rapidly changing abiotic conditions over short distances and a generally greater elevational than lateral turnover across communities (e.g., Rahbek et al. 2019).

One opportunity to address the complexity of mountain ecosystems is to adopt and adapt trait-based concepts (Suding et al. 2008; Díaz et al. 2016). Analyses of functional traits can be used for comparisons across ecosystems, which differ in taxonomic composition (Lavorel et al. 2007; Suding et al. 2008). Trait-based approaches often distinguish between response traits that determine the response of species to environmental changes and effect traits relevant for biotic processes and ecosystem functioning (Suding et al. 2008; Díaz et al. 2013; Schleuning et al. 2015). Trait-based response-effect-frameworks (REFs, Suding et al. 2008) are thus particularly suitable to explore the relationships between abiotic conditions and the diversity of functional traits in ecological communities along environmental gradients. Recently, trait-based frameworks have been proposed to quantify the variation in species interactions and their associated biotic processes and ecosystem functions (Schleuning et al. 2015, 2020). This is important, as changes in interactions modulate the response of species to climate change (Kharouba et al. 2018; Schleuning et al. 2020). Because trait-based approaches can be generalized across ecological communities, they can provide insights into how ecosystems are structured in relation to abiotic and biotic drivers (Albrecht et al. 2018) and could underpin predictive models (Shmueli 2010) of the vulnerability of mountain ecosystems to environmental changes.

Trait-based approaches have also become prominent in dynamic vegetation (DVM, Scheiter et al. 2013; Sakschewski et al. 2016) and land surface models (LSM, Wullschleger et al. 2014; Bonan and Doney 2018; Chen et al. 2020). These modeling approaches are complementary to trait-based frameworks, because they can directly link the functional community composition to ecosystem functions such as water cycling or biomass production (Díaz et al. 2019). LSMs that include leaf response traits of a few global plant functional types (PFTs) improve the plant community response with regard to ecosystem-atmosphere exchanges of matter, energy, and water compared to LSMs without considering trait diversity (Bonan et al. 2012). Further improvements are possible by replacing a-priori selected PFTs by functional trade-offs with traits related to plant anatomy, nutrient status, and physiology. The functional trade-offs determine if plant strategies (e.g., preferential allocation of carbon to fine roots) are more effective under climate change compared to other plant strategies (Pavlick et al. 2013). Recently, trait diversity calculated from global datasets (e.g., TRY, Kattge et al. 2020) was included into a DVM to assess the resilience of Amazonian rainforests to climate change (Sakschewski et al. 2016). Yet, such trait data are hardly available for highly biodiverse ecosystems in high mountains. In addition, simulations of community responses benefit from the implementation of biotic processes into LSMs (Haverd et al. 2018; Jiang et al. 2019). To improve the simulation of ecosystem-atmosphere feedbacks, LSMs must be coupled to atmospheric models (e.g., Forrest et al. 2020). While considerable progress was achieved in simulations of the community response, more independent trait data from field observations and remote sensing are needed to test the models (Hacker et al. 2018).

However, such data are in general hardly available for tropical mountain ecosystems. Based on more than 20 years of interdisciplinary research, we have collected a comprehensive stock of data on abiotic conditions, functional traits, biotic processes, and ecosystem functions in a tropical mountain rain forest of southern Ecuador to overcome this deficit (Lotz et al. 2012). Data were acquired by field surveys, ecological experiments, and remote sensing within previous research units such as the RU816 “Biodiversity and Sustainable Management of a Megadiverse Mountain Ecosystem in South Ecuador” (Bendix et al. 2013) and the interdisciplinary knowledge transfer program MRp|SE “Platform for Biodiversity and Ecosystem Monitoring and Research in South Ecuador” (Bendix and Beck 2016). The aim of the new research unit RESPECT (Environmental changes in biodiversity hotspot ecosystems of South Ecuador: RESPonse and feedback effECTs) is to complement this unprecedented long-term dataset and, based on that, to develop a framework for projecting ecosystem changes in mountain ecosystems through the combination of a trait-based REF and a new biodiversity-informed LSM. Here, we present the concept and design of the framework, new data sources, and exemplary results.

## Materials and methods

### General framework

**Fig. 1:**
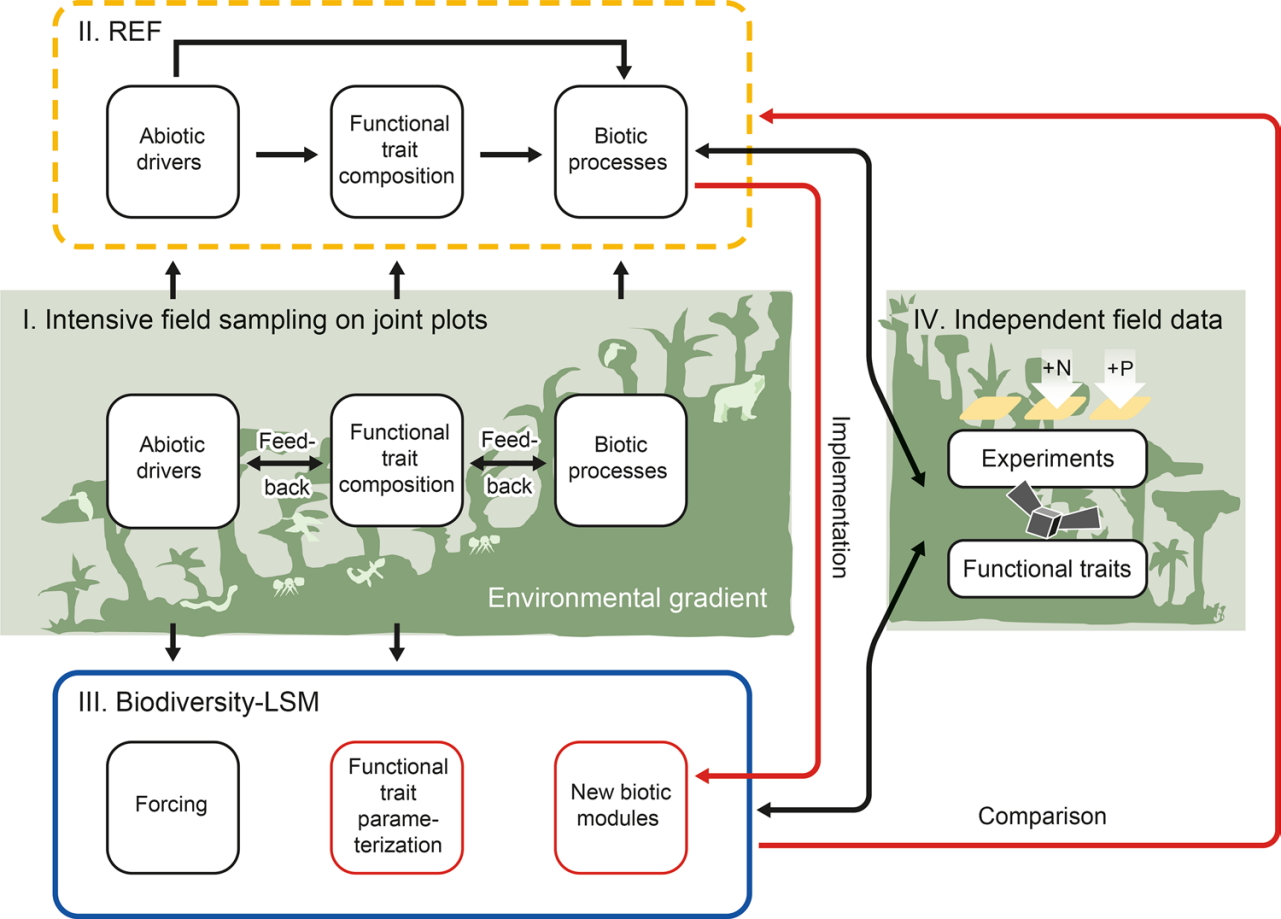
General design of the research framework: (I) intensive field sampling will provide data to (II) identify the functional trait composition and quantify biotic processes within a response–effect framework (REF) that (III) will be implemented (red arrow from II to III) into a biodiversity-LSM (land surface model). Independent test data (e.g., from experimental nutrient addition or remote sensing) (IV) are used to test REF and biodiversity-LSM results. Results are compared to assess their confidence (red arrow from III to II)

We propose a research framework for projecting ecosystem changes in mountain ecosystems that consider functional diversity and biotic processes. Such approaches are lacking due to the difficulty to collect local trait data for many taxa in highly diverse mountain ecosystems. Our framework comprises four main components (Fig. 1): (I) the collection of field data within a sampling design to quantify the variation of abiotic conditions, functional traits, and biotic processes along environmental gradients; (II) a statistical analysis of these data in a trait-based REF to identify key functional traits representative for generalizing across communities and parameterizing biotic processes; (III) the integration of key functional traits and relevant biotic processes into a biodiversity-LSM; (IV) testing the biodiversity-LSM with independent data.

The research framework can be used for (1) describing the complexity of mountain ecosystems and (2) projecting their response to environmental changes. A plot design is needed to capture the temporal response of ecosystems to environmental changes using a space-for-time approach (França et al. 2016). The REF uses data of abiotic conditions as drivers, and evaluates their direct and indirect effects on trait composition and biotic processes. The identified functional traits and their relationship to biotic processes are integrated into the biodiversity-LSM. The data on abiotic drivers or climate change scenarios are used to force, and the trait composition data to parameterize the model. Likewise, important biotic processes identified by the REF will be implemented as new modules in the biodiversity-LSM (Vinatier et al. 2016). To adapt it to the complexity of a mountain ecosystem, models for ecosystem–atmosphere transfer, vegetation dynamics, and soil water need to be coupled (Davies-Barnard et al. 2020). Generating a biodiversity-LSM does not strive to represent all species, which is impossible in a tropical biodiversity hotspot with more than 100 tree species ha^−1^. Instead, it aims at representing the trait diversity and relationships between traits across species to capture relevant biotic processes (e.g., herbivory and seed dispersal). The quality of the biodiversity-LSM is tested by independent data from our plots, remote sensing, or collected in ecological field experiments.

### An example for mountain ecosystems

We use a tropical mountain forest located in the SE-Andes of Ecuador (Beck et al. 2019) with its unique biodiversity (Homeier et al. 2010) as a showcase for our framework, which is representative to other high mountain area. The responses of this ecosystem to climate change is due to its complexity not well understood (Morueta-Holme et al. 2015), because climate change not only leads to warmer temperatures (Peters et al. 2013), but also to changing rainfall (e.g., Buytaert et al. 2010), associated declines in soil moisture, and changing nutrient deposition (Wilcke et al. 2013, 2019). Additionally, the natural forest is threatened by deforestation (Curatola Fernández et al. 2015; Tapia-Armijos et al. 2015). The overall aim of developing the research framework is to project the response of the tropical mountain forest and its anthropogenic replacement ecosystems, in this case pasture, to climate change. We focus on two ecosystem target functions, i.e., (1) biomass production and (2) water fluxes. Biomass production is important to produce pasture and timber products to cover the livelihoods of the local population (Knoke et al. 2014, 2016). Furthermore, mountain forest ecosystems are important for carbon sequestration to mitigate CO_2_-induced climate change. The water exchange between ecosystem and atmosphere (in form of latent heat flux by evapotranspiration ET) is an essential proxy for biologically induced changes in the ecosystem’s water balance due to climate and land-use changes (e.g., Silva et al. 2017). Because the quantity of ET determines how much groundwater and overland flow are generated, its response to environmental change is a key for potable water supply and hydropower generation (Carvajal et al. 2019). Thus, our main objective is how these two target functions are affected by climate and land-use changes through changes in community composition in respect to response and effect traits. Climate and land-use change have been shown to reduce trait diversity in our area. However, trait diversity stabilizes biotic processes and ecosystem functions by functional redundancy in the community (Santillán et al. 2018). Functional traits also mediate biotic processes, such as herbivory and seed dispersal (Werner and Homeier 2015; Quitián et al. 2019). Thus, we hypothesize that including key functional traits and biotic processes in a state-of-the-art LSM will result in a biodiversity-LSM that realistically projects changes of the target functions and the resistance of complex ecosystems under climate change.

### Intensive field sampling on joint plots

**Fig. 2:**
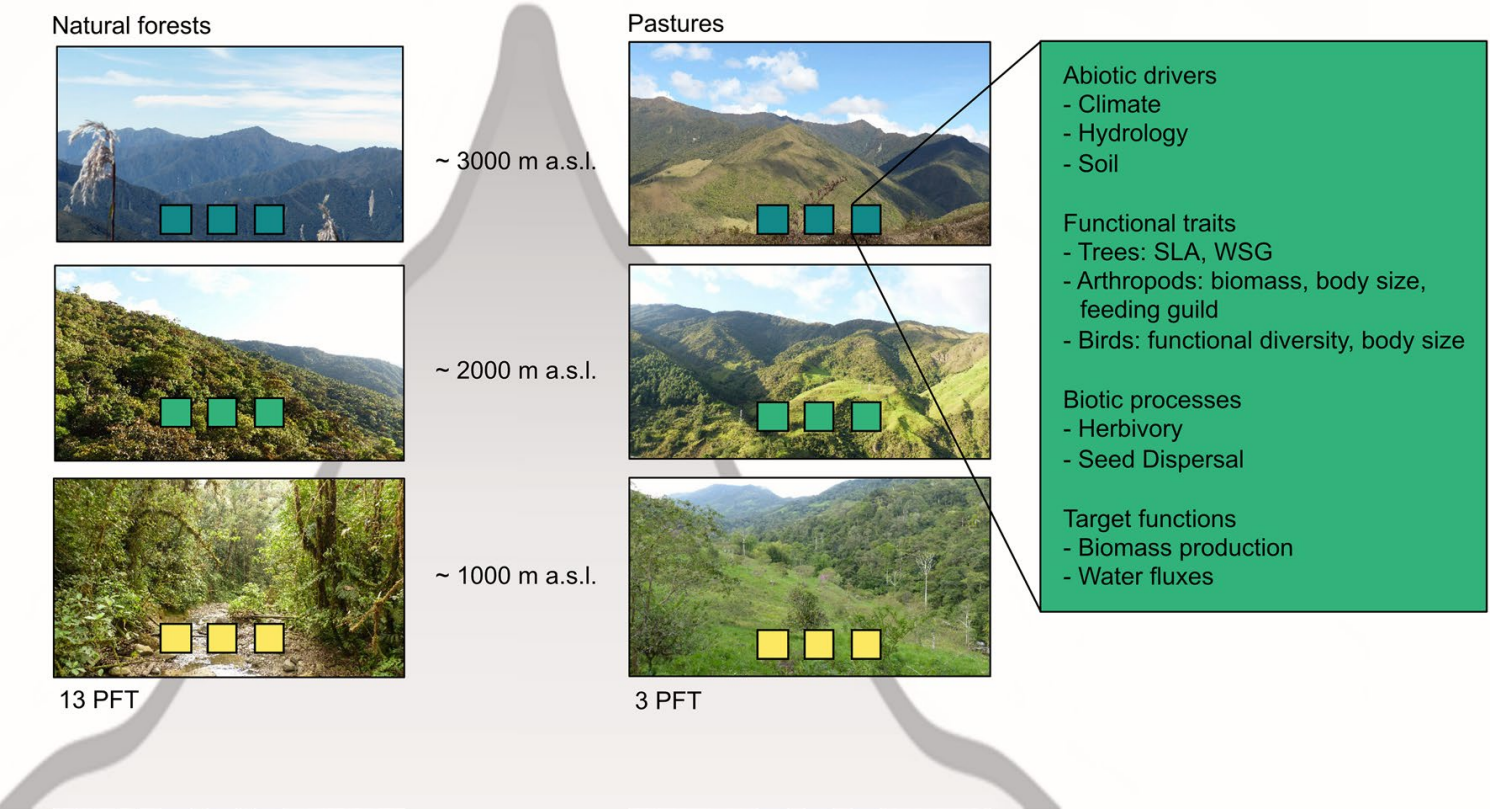
The coherent plot system comprised 18 one-hectare plots that were distributed along an elevational gradient in natural forests and pastures. On the plots, abiotic data, functional traits (SLA specific leaf area, WSG wood-specific gravity), tree abundance, and biotic process data were recorded on predetermined plant species for a-priori defined plant functional types (PFT). Independent field and remote-sensing data were recorded for the two target functions (Examples in the non-comprehensive list to the right.)

**Fig. 3:**
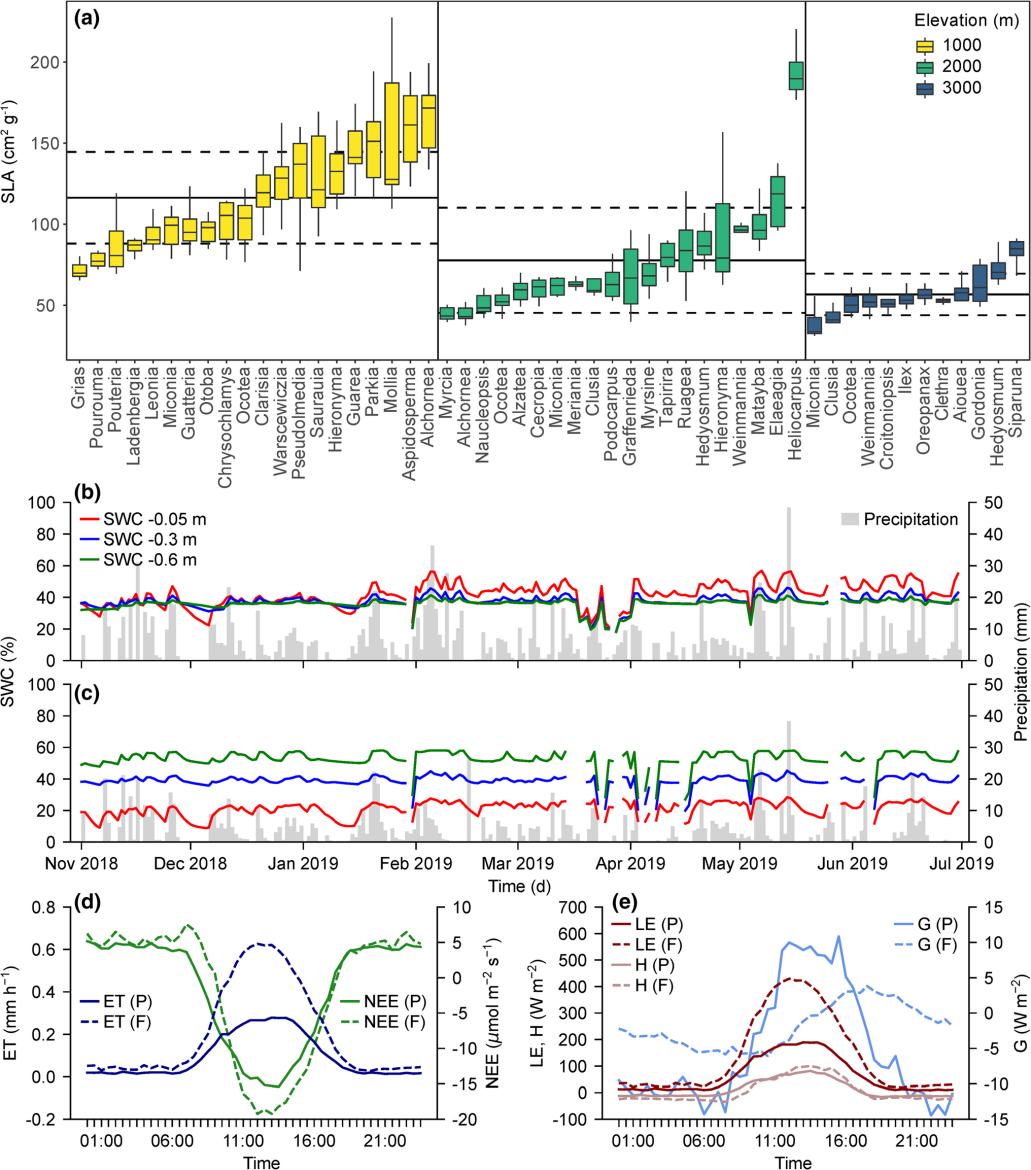
The approach relies on three data types: **a** trait data, used to describe trait diversity in the biodiversity-LSM (land surface model) and for REF (response–effect framework) analyses. SLA (specific leaf area) values of 52 tree species (8 replicates) revealed on average higher values at the lowest altitude (1000 m a.s.l.) (full gray line), at a high average and interspecific variation along the entire elevation gradient (dashed lines). Many other functional traits were correlated with SLA, so we used it as one key trait (III Figs. 1, 5) for the REF and the biodiversity-LSM. **b**–**c** Data from hydro-climate stations (**b** pasture, **c** forest; at 2000 m a.s.l.) such as soil water content (SWC), showing distinct differences between forest and pasture, and precipitation were used for model forcing and as covariates in the REF analyses (II and III Fig. 1). **d**–**e** Independent Eddy covariance flux data (**d** evapotranspiration ET, net-ecosystem exchange NEE) and measured surface energy fluxes (**e** sensible H, latent LE, and ground heat flux G) were used for testing of the biodiversity-LSM (IV in Fig. 1), showing higher ET over the forest (F; blue dashed line) during daylight than over pastures (P; full blue line) and differences in NEE (green lines)

We implemented a joint plot design, covering the elevational and land-use gradients in our study area, with 18 one-hectare plots distributed from 1000 to 3000 m a.s.l. in natural forests and pastures (Fig. 2). We collected data on abiotic conditions, functional traits, and biotic processes (Table 1). Initially, we selected a-priori defined PFTs based on specific leaf area (SLA) and wood-specific gravity (WSG), which are not only relevant for ecosystem processes in our study area (Homeier et al. 2010; Báez and Homeier 2018), but have also been used to estimate biomass production on a global scale (Díaz et al. 2016). Analyses of these traits and relationships between traits revealed that trait variation is rather continuous in the natural forest ecosystem. We therefore used the full variation of traits in the biodiversity-LSM instead of discrete PFTs normally used in LSMs. Nevertheless, an a-priori PFT definition was important for sampling functional traits of representative tree species, as sampling all of the > 1000 tree species occurring in the area would have been probative time consuming. For four representative tree species of each PFT (52 species and 13 PFTs), we quantified trait data (e.g., SLA, Fig. 3a) which are related to both target functions in REF analyses and LSMs (e.g., Paulick et al. 2017; Table 1). Furthermore, we collected data on abiotic drivers with a high temporal resolution on the plots (Fig. 3, Table 1). Selected abiotic drivers (e.g., irradiance) and functional traits (e.g., photosynthetic quantum use efficiency) were collected from canopy towers that are available for one plot per elevation in the natural forest. Besides the functional trait data for PFTs, we collected species composition and functional trait data of arthropods and birds, such as body size, feeding guild, and morphometric properties (e.g., Santillán et al. 2018; Quitián et al. 2019; Table 1).

**Table 1:** Abiotic drivers, functional trait data, biotic processes, and independent test data for the two target ecosystem functions (TF)

Abiotic drivers	Functional traits	Biotic processes	Independent test data
Climate variables	Leaf optical traits (radiances, indices)	NPP	*TF water fluxes*
Atmospheric deposition	Leaf N (C/N), leaf N fraction in RubisCO	Tree growth	Water/energy fluxes (Ecov)
Soil physics	Root (C/N)	Root water uptake	ET (RS, experiments)
Soil water and leaching	Leaf chlorophyll content	Tree water conductance	
Soil chemistry (mainly N, P)	SLA	Sapflow	*TF biomass production*
	Leaf thickness	Photosynthesis	Carbon flux, NEE (Ecov)
	Leaf palatability	Plant recruitment	Ecosystem respiration Reco (Ecov)
	Fruit and seed traits	Herbivory	Biomass and productivity (RS, experiments)
	WSG	Seed dispersal	LAI, VIs (RS)
	Body size and shape, morphometric traits (arthropods, birds)		
	Feeding guild (arthropods, birds)		
	Water use efficiency (WUE)

### The biodiversity-land surface model (LSM)

**Fig. 4:**
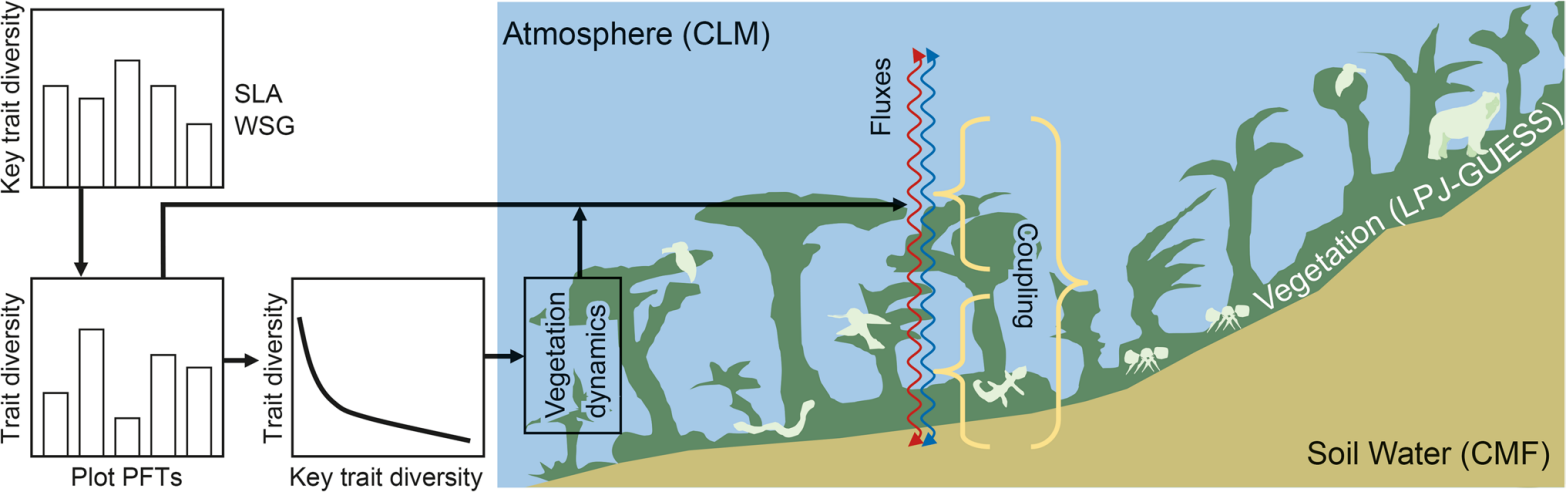
The biodiversity-LSM (land surface model) includes LPJ-GUESS as the core model, where the essential biotic processes have been or will be implemented, CMF (Catchment Modeling Framework), offering the best representation of soil water processes and CLM (Community Land Model), and calculating water and energy fluxes between the ecosystem and the atmosphere. LPJ-GUESS and CLM are using soil water from CMF. CLM is parameterized by the change of vegetation and community trait composition from LPJ-GUESS. For the natural forest, all PFT trait data of the plots are used in LPJ-GUESS as a trait diversity continuum. To simplify parameterization in the highly diverse mountain forest, trade-off relationships between key (SLA = specific leaf area; WSG = wood-specific gravity) and dependent traits (e.g., leaf C:N ratio) are derived (Fig. 5 for details)

**Fig. 5:**
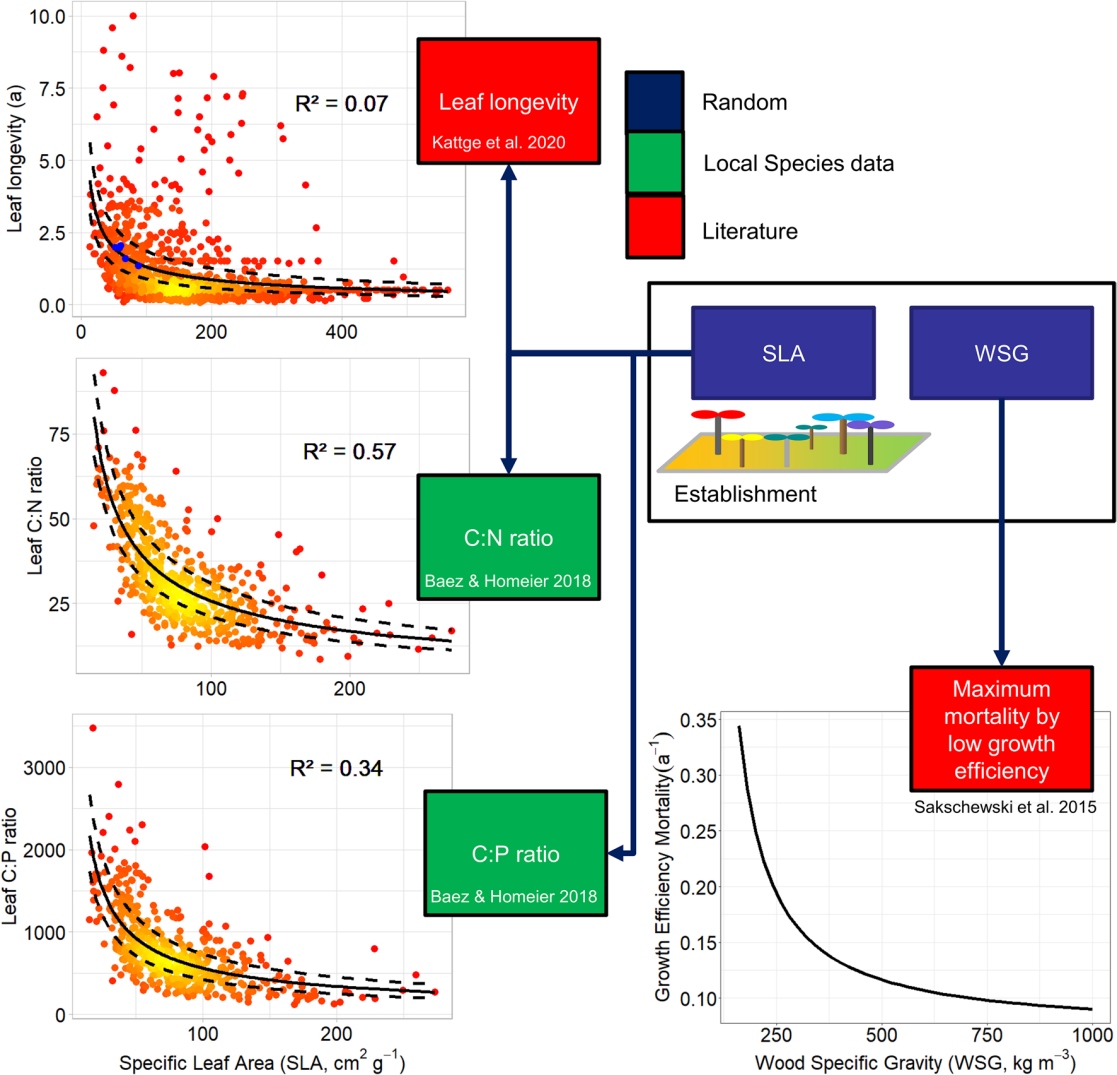
Strategy for implementing trait variability in our version of LPJ-GUESS to consider the high plant diversity for the model, based on local trait and literature data. Random independent key traits (dark blue) are drawn from a uniform distribution at establishment, from which several other dependent traits (green, red) are calculated using trade-off–relationships (left panels using the key trait specific leaf area; lower right by applying wood-specific gravity). Solid curves depict average fits; dashed lines depict standard errors

Instead of using an existing state-of-the-art LSM, we coupled and improved three models which cover the relevant compartments of the ecosystem to establish the biodiversity-LSM (Fig. 4): The dynamic vegetation model Lund–Potsdam–Jena General Ecosystem Simulator LPJ-GUESS (Smith et al. 2011, 2014), the Catchment Modeling Framework CMF (Kraft et al. 2011; Windhorst et al. 2013), and the Community Land Model CLM (Silva et al. 2012; Hurrell et al. 2013). The LPJ-GUESS model, where trait composition and biotic processes are implemented, includes a detailed representation of tree population dynamics and the simulation of individual trees. In our new version, each individual has a specific trait value and the community trait composition emerges via ecological sorting (individuals with the best adapted traits outcompete other species; Sakschewski et al. 2015). To reduce the complexity of the highly diverse ecosystem, we drew randomly key traits for each established individual from a uniform distribution (Fig. 5). We used SLA and WSG, traits that are closely correlated to other traits (Fig. S1 and Table 1) needed for the biodiversity-LSM. Correlations can be derived using trade-off relationships to the local key traits (Fig. 5). The first implementation uses the dependent local traits: (1) carbon-to-phosphorus (C:P) (2) and carbon-to-nitrogen (C:N) concentration ratios in plant tissue related to the key trait SLA. Other relationships (e.g., leaf longevity) are still relying on global trait databases (TRY; Kattge et al. 2020) or literature surveys, but will be also replaced by local plot data. Regarding biotic processes, a module herbivory is implemented as energy transformation through local herbivore communities following Wiegert and Petersen (1983). Main elements of the module are (1) the leaf input to herbivores by removing a percentage of the total individual plant leaf mass and (2) respiration losses of leaf carbon through herbivore metabolism. Seed dispersal is the other process of biotic interaction which is generally implemented in the biodiversity-LSM. Both modules, however, must be locally adapted with local plot data and results from the REF. The final biodiversity-LSM shall be used to project the resistance of the ecosystem against environmental changes by forcing the model with scenarios of climate change for both, the natural forest and the pasture system. The comparison of simulations will unveil the combined effects of climate and land-use changes on the resistance of the target functions.

## Results and conclusion

### Response–effect framework (REF)

**Fig. 6:**
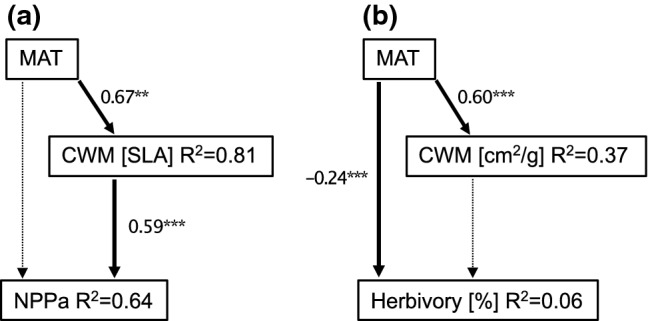
Structural equation model showing direct and indirect effects of mean annual temperature (MAT), community-weighted mean of specific leaf area (CWM [SLA]), or individual SLA (cm^2^/g, measured for 355 trees with 4–8 individuals per species) on **a** aboveground biomass production (NPPa, estimated using stem increment and litter production) and **b** herbivory [%]. Dashed arrows depict omitted effect in partial mediated models. Effect sizes for significant effects are given next to arrows with asterisks demarking the significance level (**> 0.001 < ***> 0.000). For each endogenous variable, the relative amount of explained variance is given. We used the Chi^2^-difference-test to assess whether our saturated model is more or less supported than the partial mediation model. For both models, the Chi^2^-difference-test supported the partial mediation model (NPP: Chi^2^ = 5.25; *df* = 2; *p* value = 0.072; herbivory: Chi^2^ = 5.99; *df* = 2; *p* value = 0.050, with similar results in the full models)

In two examples, we used the REF to assess the relevance of trait data from the actual plot system for quantifying biotic processes that are important for our target functions (e.g., NPP). Such analyses were done to identify functional traits that can be generalized across communities and are relevant to parameterize biotic processes. We used community-weighted mean (CMW) trait values (plot-level trait values weighted by species abundance) of specific leaf area (SLA) to analyze their importance for the ecosystem function biomass production (Fig. 6a) as well as trait values (SLA) of the above-mentioned individual trees of 52 species within plots to unveil their importance for the biotic process herbivory (Fig. 6b). We used structural equation models to estimate direct and indirect effects of abiotic drivers (mean annual temperature MAT), functional trait diversity (SLA) on biotic processes (herbivory), or our target function (NPP). We found a direct and positive effect of increasing temperatures on the CWM of SLA as well as a direct positive association between the CWM of SLA and aboveground NPP (Fig. 6a). Similarly, we observed a direct and positive effect of increasing temperatures on the SLA of the 52 tree species. In contrast, we found a direct negative effect of increasing temperature on herbivory (leaf area loss of the 52 study species). We did not see an association between SLA and herbivory of tree individuals (Fig. 6b). These findings highlight that effects of abiotic drivers can be both direct and indirect depending on the functional trait composition.

### Performance of the biodiversity-land surface model

**Fig. 7:**
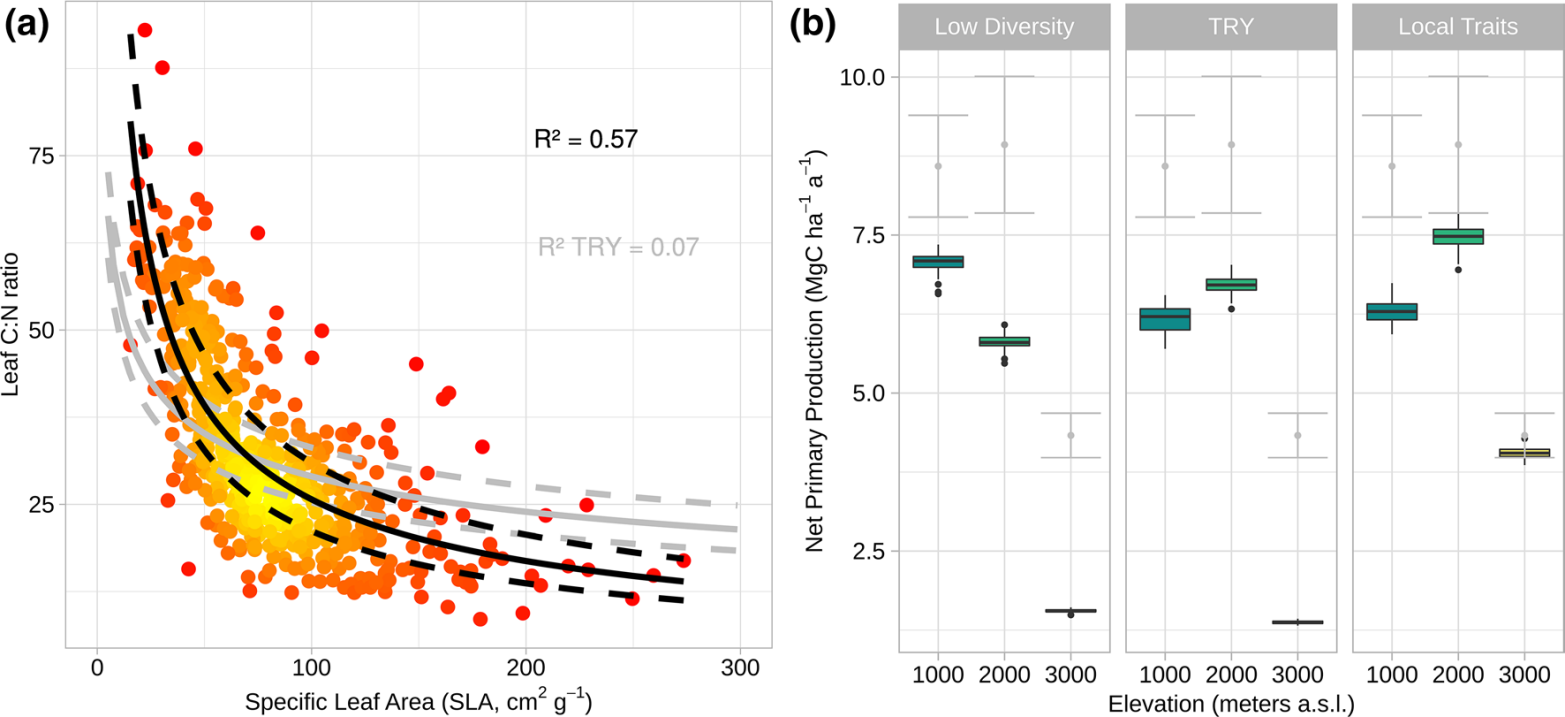
Examples for independent testing of the local adaptation of LPJ-GUESS by simulating net primary production (NPP) for three elevation sites. **a** Global (TRY) and local SLA to C:N ratio relationships where the solid curves represent the average fits, dashed lines the standard error. **b** The low diversity simulation (left) uses individuals with a constant average SLA. Furthermore, simulations with global (center) and local (right) SLA to leaf carbon-to-nitrogen ratio (C:N) relationships are compared. The test against independent average NPP data (gray points/lines) showed best performance using local trait relationships. The model was forced by local temperature, precipitation, radiation, and nutrient input values (i.e., deposition and weathering rates) and was run for 700 years on a 10-hectare area, with the results derived as an average of the last 200 years

Testing the model is and will be done with independent data that are not used for parameterizing the model (Fig. 1IV.). This can be observational data of the target functions from the plots (e.g., ET in Fig. 3d), field data of which were collected during previous research (e.g., biomass; Wallis et al. 2019), and data of ecological experiments (e.g., nutrient manipulation experiments; Homeier et al. 2017). Using such data (here NPP from Leuschner et al. 2013), we tested the improvement of the biodiversity-LSM at its current state of development (Fig. 4, 5). For this, we defined the minimum and maximum ranges of the key trait SLA (Fig. 7a), and their relationships with other traits using the local trait data (here leaf C:N ratio). In a first scenario (Fig. 7b, left), we run the simulations using the low diversity mode, in which the independent traits were fixed to an average value for the elevational gradient. This represents the approach commonly used by state-of-the-art LSMs, which do not implement trait diversity. The results underestimated NPP, especially for the highest elevation site. In contrary, the model with local trait diversity driven by local climate, nutrient availability, and trait relationships was able to correctly predict the target function NPP (Fig. 7b right). Nutrient limitation of both, nitrogen and phosphorus, was identified as a key abiotic driver to represent the observed changes of biomass production. When running the same simulations using globally defined trait relationships derived from the TRY database (Kattge et al. 2020), model performance was substantially reduced for the target function biomass production (NPP, Fig. 7b center). The results demonstrate the importance of including local trait diversity to gain improved biodiversity-LSMs.

## Conclusion

The lack of available local data on abiotic drivers, functional traits, and the missing integration of biotic processes into LSMs have hampered scientific progress in projecting community answers to global change, particularly in highly diverse ecosystems. We propose that integrating detailed data on functional trait diversity and biotic processes of complex ecosystems into statistical and LSM modeling approaches will advance understanding of the functional importance of biodiversity and projections of the consequences of global change for highly diverse mountain ecosystems. The approach centered on the biodiversity-LSM also allows to project ecosystem–atmosphere feedbacks. Projections on these feedbacks, e.g., water vapor exchange at the ecosystem–atmosphere interface or carbon sequestration through biomass growth under climate change, require a local representation of dominating trait composition changes and biotic processes. These local feedbacks determine whether ecosystem changes contribute to an acceleration or mitigation of global climate change impacts on ecosystems and are thus essential in the biodiversity-LSM part of our framework. Thus, we provide the conceptual workflow and framework that can be used to make projections of likely responses of tropical mountain ecosystems to global change scenarios. We developed this research framework from our perspective on tropical mountain rainforests, but also point out that it can be used and developed further for a wide range of ecosystems. To transfer the research framework to other ecosystems, our examples showed that a dataset of locally collected essential variables is necessary. These comprise abiotic drivers such as temperature that depict the environmental gradient for the REF as well as for model forcing, plant and animal functional traits, vegetation characteristics, and quantitative data on biotic processes that are relevant for the respective target functions of the ecosystem. We believe that such integrated approaches, combining field measurement on functional traits, biotic processes, as well as ecosystem–atmosphere exchanges with statistical and process-based modeling are necessary to fundamentally advance our ecosystem understanding.

## Supplementary Information

Below is the link to the electronic supplementary material.

Supplementary file1 (PDF 216 KB)
