# Supplementary material for: A research framework for projecting ecosystem change in highly diverse tropical mountain ecosystems
Source: Oecologia. 2021 Jan 30;195(3):589–600. doi: 10.1007/s00442-021-04852-8 (PMC7940296; doi:10.1007/s00442-021-04852-8)
Supplement: Supplementary file 1 — Supplementary file1 (PDF 216 KB) [file 442_2021_4852_MOESM1_ESM.pdf]

Supplementary material

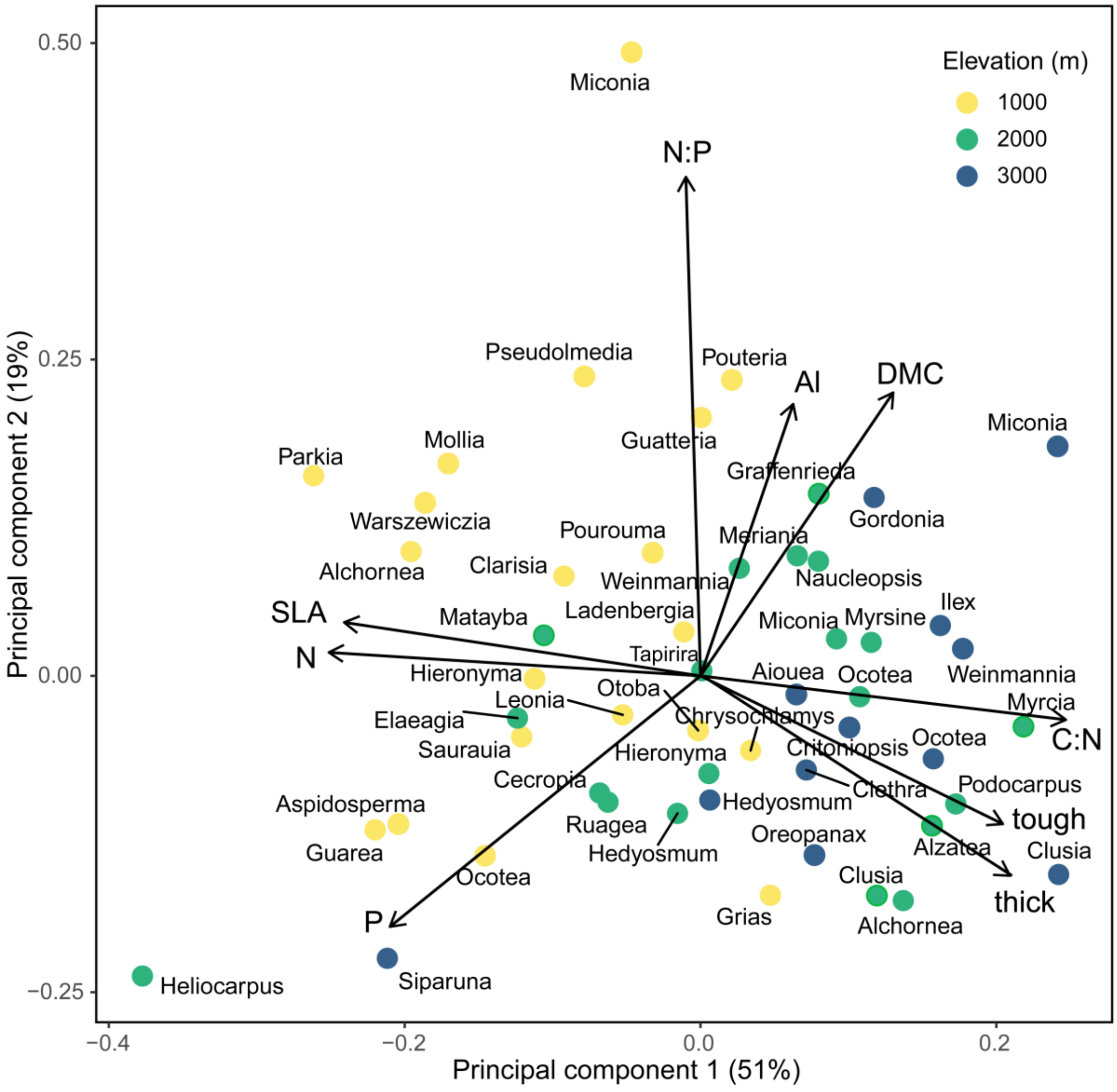

Fig. S1 Descriptive principal component analysis based on the correlation matrix across species to illustrate relationships between traits. This analysis is based on nine leaf traits (AL refers to foliar Al, C:N to foliar C/N ratio, DMC to dry matter content, N to foliar N, N:P to foliar N/P ratio, P to foliar P, SLA to specific leaf area, thick to leaf thickness, tough to leaf toughness) recorded for 52 tree species at three elevation levels (colour as in Fig. 2, 3a and 7b). For simplicity we have not considered the phylogeny of the tree species.
